# A review on tumor treating fields, a novel modality in cancer treatment

**DOI:** 10.1080/07853890.2021.1896891

**Published:** 2021-09-28

**Authors:** Nichal Gentilal, Pedro Cavaleiro Miranda

**Affiliations:** Institute of Biophysics and Biomedical Engineering, Faculdade de Ciências, Universidade de Lisboa, Lisboa, Portugal

## Abstract

**Introduction:**

Glioblastoma Multiforme (GBM) is one of the deadliest tumours that appears in the brain and it is characterised by its aggressiveness and by a very short overall survival (OS) [1]. Despite all the efforts that have been put into trying to improve treatment outcomes, GBM patients’ prognosis is still very poor. In the last couple of years, a new technique was developed to target tumoral cells. Tumour Treating Fields (TTFields) are intermediate-frequency (100–300 kHz) alternating electric fields (1–3 V/cm at the tumour bed) that can affect cells mitosis during metaphase and cytokinesis. Results from clinical trials [2,3] showed that this technique can lead to an increased OS both in newly diagnosed and recurrent patients. Our aim is to address the main findings regarding this therapy and discuss future challenges based on a literature review of the papers published since this technique was first reported in 2004 to the present date.

**Materials and methods:**

This literature review was performed considering only publications made in journals with impact factor in the areas of interest (biomedical engineering and oncology). Appropriate keywords (TTFields, alternating electric field therapy, glioblastoma) were used to filter the results and select the relevant publications in Pubmed and Web of Science until May 2019. The total number of papers analysed was 29.

**Results:**

Results from clinical trials showed that a minimum daily usage of 18 h can improve the OS, while other studies showed that switching the direction of the applied field between two perpendicular orientations alternately increases the number of cells that are affected by this technique. Up until now, only skin dermatitis was reported as a side-effect due to the usage of a hydrogel between the scalp and the electrodes. Computational studies allowed to predict the electric field in the brain during therapy and subsequent studies proved that the uncertainty regarding biological tissues parameters (e.g.: electrical conductivity) might have a significant impact on these predictions. Furthermore, the best electrodes positioning on the scalp and the impact of removing a part of the skull to enhance the electric field magnitude in the tumour are some important topics that are being discussed.

**Discussion and conclusions:**

Despite the quick developments since it was first reported, TTFields investigation is still at its beginning. Some current research includes the thermal impact of this therapy and the study of its applicability in other types of cancer such as non-small cell lung cancer (NSCLC) and pancreatic and ovarian cancers. Nonetheless, this technique proved to be a very useful therapy in the treatment of GBM and all the results obtained so far point out to TTFields being an excellent fourth modality to fight cancer.
